# Multiple Factors Affect Socioeconomics and Wellbeing of Artisanal Sea Cucumber Fishers

**DOI:** 10.1371/journal.pone.0165633

**Published:** 2016-12-08

**Authors:** Steven W. Purcell, Poasi Ngaluafe, Simon J. Foale, Nicole Cocks, Brian R. Cullis, Watisoni Lalavanua

**Affiliations:** 1 National Marine Science Centre, Southern Cross University, Coffs Harbour, New South Wales, Australia; 2 Ministry of Agriculture & Food, Forests and Fisheries, Nuku’alofa, Tonga; 3 College of Arts, Society and Education, James Cook University, Townsville, Queensland, Australia; 4 School of Mathematics and Applied Statistics, National Institute for Applied Statistics Research Australia, Faculty of Engineering and Information Sciences, University of Wollongong, Wollongong, Australia; 5 Partners in Community Development Fiji, Suva, Fiji; 6 Wildlife Conservation Society, Suva, Fiji; Leibniz Center for Tropical Marine Ecology, GERMANY

## Abstract

Small-scale fisheries are important to livelihoods and subsistence seafood consumption of millions of fishers. Sea cucumbers are fished worldwide for export to Asia, yet few studies have assessed factors affecting socioeconomics and wellbeing among fishers. We interviewed 476 men and women sea cucumber fishers at multiple villages within multiple locations in Fiji, Kiribati, Tonga and New Caledonia using structured questionnaires. Low rates of subsistence consumption confirmed a primary role of sea cucumbers in income security. Prices of sea cucumbers sold by fishers varied greatly among countries, depending on the species. Gender variation in landing prices could be due to women catching smaller sea cucumbers or because some traders take advantage of them. Dissatisfaction with fishery income was common (44% of fishers), especially for i-Kiribati fishers, male fishers, and fishers experiencing difficulty selling their catch, but was uncorrelated with sale prices. Income dissatisfaction worsened with age. The number of livelihood activities averaged 2.2–2.5 across countries, and varied significantly among locations. Sea cucumbers were often a primary source of income to fishers, especially in Tonga. Other common livelihood activities were fishing other marine resources, copra production in Kiribati, agriculture in Fiji, and salaried jobs in New Caledonia. Fishing other coastal and coral reef resources was the most common fall-back livelihood option if fishers were forced to exit the fishery. Our data highlight large disparities in subsistence consumption, gender-related price equity, and livelihood diversity among parallel artisanal fisheries. Improvement of supply chains in dispersed small-scale fisheries appears as a critical need for enhancing income and wellbeing of fishers. Strong evidence for co-dependence among small-scale fisheries, through fall-back livelihood preferences of fishers, suggests that resource managers must mitigate concomitant effects on other fisheries when considering fishery closures. That is likely to depend on livelihood diversification programs to take pressure off co-dependent fisheries.

## Introduction

### Small-scale fishery resources and livelihoods

Artisanal or small-scale fisheries play a crucial role as a source of livelihoods, food security and income for millions of people in tropical countries [1, 2], but differential benefits to fishers might be affected by a range of factors. Understanding the socioeconomic characteristics of fisheries is important for planning regulatory measures to improve resource sustainability [3]. Such information could assist in designing development programs or interventions to optimise economic benefits for local communities. Income from coastal resources derives from selling fish and invertebrate catches to local or export markets [4–6]. However, factors including a lack of knowledge of the market price for key species, or poor handling and processing, may influence prices received by local fishers [5, 7].

Both men and women are involved in inshore fisheries for fishes and invertebrates [8, 9]. While there is increased recognition of women’s contribution in the fisheries sector [8, 10], gender inequity persists in various facets of fisheries [8, 11] and is often overlooked in planning of fisheries management and development [9, 12]. In particular, there are few data from developing countries of comparative economic gains for men and women in fisheries [12]. Understanding socio-economic disparities in fisheries is a first step to addressing gender inequities [12], such as through targeted training programs.

### Livelihood activities and wellbeing

Small-scale fishers in the Indo-Pacific are considered to have an extremely marginal livelihood [13]. The collapse or closure of economically valuable artisanal fisheries, such as sea cucumbers, has often been experienced as comparative austerity for many islander groups [14–17], because most available alternative sources of income entail unattractive returns on labour. However on islands where fertile land is comparatively abundant, income from export-commodity agriculture (vanilla, cocoa, coffee, palm oil, virgin coconut oil) and urban food markets can be more enduring and lucrative, depending on market access [18]. Production of copra (dried coconut flesh) has been ephemeral among island nations due to fluctuations in commodity prices for coconut oil, and the buying price for copra is often unattractive for villagers without subsidies [19, 20]. In rural Pacific Island villages, other common livelihood options include handicrafts, such as mat making from *Pandanus* or tapa (barkcloth), small artisanal businesses, and remittances from relatives working overseas [21].

Wellbeing in fisheries relates not only to the health of fishery workers but also to whether human needs are met such that one can enjoy a satisfactory quality of life [22]. Fisher satisfaction is influenced by income and happiness, as well as non-monetary benefits such as adventure and self-actualisation [23–25]. Dissatisfaction impacts on fishers health, both physical and psychological [24, 26], and may erode relationships between fishers and management institutions [27, 28]. Comparatively few studies have assessed wellbeing and income satisfaction in small-scale fisheries, which employ more than 90% of fishers worldwide [29]. Understanding some of the factors impacting on fisher wellbeing offers an opportunity to target training and development programs for fisheries.

### Artisanal Pacific Island fisheries

Coastal communities in Oceania depend heavily on nearshore small-scale fisheries for subsistence protein and cash income [30]. In terms of food security, finfish contribute significantly while a number of other fisheries, such as sea cucumbers, trochus and aquarium ornamentals, provide income streams and contribute to national export revenue [2, 31].

Many Pacific Islands populations decreased in the 19^th^ Century due to introduced diseases, but then increased rapidly during most of the 20^th^ Century [32]. This population pressure, combined with the rapid expansion of markets, and material aspirations, have resulted in widespread overharvesting of commodity fisheries [18, 33–36].

Sea cucumbers are harvested from coral reefs and coastal habitats from practically all tropical countries and exported to Asian seafood markets in the dried form, called ‘bêche-de-mer’ or ‘trepang’ [37, 38]. Owing to high market demand from Asia, and high shipping costs that constrain reef fish exports, sea cucumber fisheries are claimed to be the second-most valuable export fishery in the South Pacific, behind tuna [7]. Among export commodities of Pacific Islands, more live tonnage in sea cucumbers is extracted and traded annually than all other reef fisheries combined [30]. Ineffective management and overfishing has led to recent closures of commercial sea cucumber fisheries in Vanuatu, Solomon Islands, Papua New Guinea and French Polynesia [39, 40], and most recently (in 2015) in Tonga and Kiribati. At the time of this study, Fiji, Kiribati, Tonga and New Caledonia were among the few countries still exporting sea cucumbers and these fisheries had different fishery contexts and management systems [41].

Pacific Islanders have been active traders since pre-colonial and early colonial times [42, 43]. Fishing for sea cucumbers in Pacific Island countries has been recorded for at least 170 years for both commercial and subsistence purposes [44–47]. Outside the south-east Asian countries, subsistence consumption of sea cucumbers is insignificant in most regions of the world [48–50]. Pacific Islands are a noted exception, where subsistence consumption is known to occur in at least 11 countries [51], but the importance to diets has rarely, if ever, been quantified.

### Study purpose and significance

The study aim was to assess factors that affect subsistence consumption, sale prices, income satisfaction, and livelihood diversity of sea cucumber fishers among four Pacific Island countries: Fiji, Kiribati, Tonga and New Caledonia. These countries are from the three cultural regions of Oceania: Melanesia, Micronesia and Polynesia. Fishers in these countries predominantly used small-scale and artisanal fishing gears and practises [41]. The standardised data collection allowed for tests across multiple spatial scales (countries, locations, and villages) and among other factors including gender, age and experience of fishers.

Data collection was part of other studies, which also examined fishing activities of the fishers and the methods used for postharvest processing of the sea cucumbers [41, 52]. This study represents the first multi-country socioeconomic comparison of sea cucumber fishers in the primary literature. Understanding factors that influence socioeconomics and wellbeing of fishers is essential for planning management measures and livelihood support programmes. Our analyses highlight multi-level variations in socioeconomic metrics, which are relevant to understanding other small-scale fisheries globally.

## Materials and Methods

### Study locations and period

**Fig 1:**
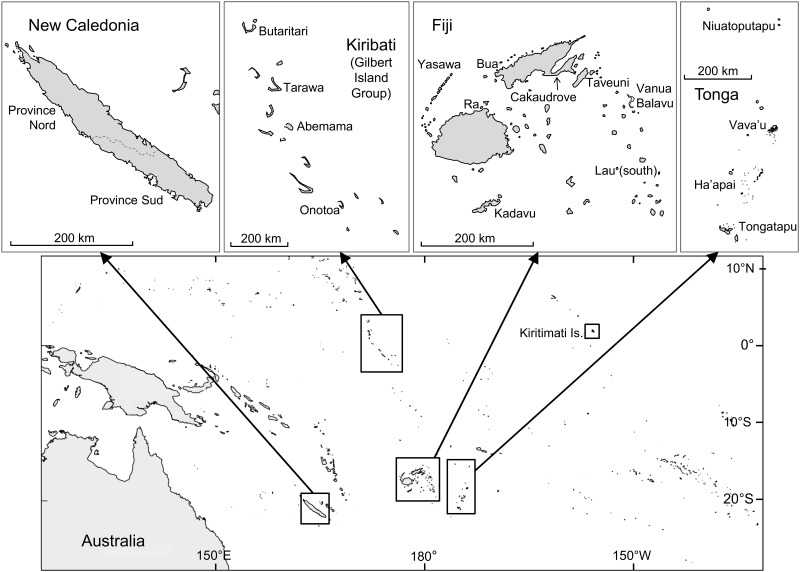
Countries and locations in the study. Map of the central-western Pacific showing the four study countries. Individual maps show the study locations within each country; Kiritimati Island in the Line Islands Group of Kiribati was also a study location.

The study locations and data collection methods were as described by Purcell et al. [41], and briefly outlined herewith. The study was conducted in four Pacific Island countries: Fiji, Kiribati, New Caledonia and Tonga (Fig 1). Based on advice from national or provincial fishery authorities, we selected locations (provinces or island groups) within each country where sea cucumber fishing was taking place. For Fiji, Kiribati, Tonga and New Caledonia, 8, 5, 4, and 2 locations were selected, respectively (Fig 1). Within locations, we generally visited 3–6 villages that were known to have fishers who collected sea cucumbers (whether occasionally or regularly).

Duration and period of the data collection differed among countries, owing to the scope determined by research funding. Surveys and interviews with fishers in New Caledonia were conducted from Aug–Dec 2007, and we had not included some questions (e.g. sale prices) that were later posed to fishers in Kiribati, Tonga and Fiji [53]. Surveys and interviews in Tonga and Kiribati were completed during 2011, and those in Fiji were conducted during 2014. Although fishing and exports in these countries operate independently, we interpret inter-country comparisons tentatively due to the different years of data collection. National fishery management regulations differed among the four countries [41], and community-based management was a minor part of the fisheries, or non-existent.

### Survey methodology and data collection

Data were collected through questionnaire-based interviews of fishers who had recently harvested sea cucumbers. Within each village, an average of five fishers were found for interviews using the ‘snowball’ technique and key informants, irrespective of fisher ages, fishing mode, frequency of fishing trips and catches. To improve the gender-inclusiveness of data [54], women fishers were interviewed where possible. The number of fishers interviewed in each country depended on funding, human resource constraints and the fishery context. For example, there were fewer than 100 sea cucumbers fishers in New Caledonia at the time of the surveys, but more than 7,000 in Fiji.

Interviews of 40–60 min were conducted predominantly at fishers’ homes or in open places within villages. We read questions from a semi-structured, standardised, questionnaire [41, Appendix A] such that questions used in this study were identical across interviews and countries. We repeated or asked questions in alternative ways to ensure comprehension, and used photographic identification sheets to confirm local species names with fishers. Interviews were mostly conducted by a national researcher, and an interpreter was used when a foreign researcher conducted interviews.

Using the questionnaires, we recorded each fisher’s age, gender, years of experience fishing sea cucumbers, and posed questions about subsistence consumption, livelihood diversity (number of income streams) and fall-back income streams if the fisher was forced to exit the fishery (e.g. through decimation of stocks by disease, or instigation of a fishery moratorium). As one measure of wellbeing, we asked fishers whether they were very satisfied, satisfied, unsatisfied or very unsatisfied with the income they make from fishing and selling sea cucumbers, corresponding to respective data ranks of 2, 1, -1, and -2. We asked fishers if they were happy to tell us prices for sea cucumbers they sold, and we recorded the form (fresh, dried, salted, first-cooked) the animals were sold and the unit of sale (per piece, per kg, per bucket) for each species sold. Sea cucumbers were sold by fishers to buyers (middlemen or exporters) either as fresh product, sold mostly by the piece, or dried bêche-de-mer, sold by the kilogram. When fishers sold both fresh and dried sea cucumbers, we recorded data on both forms. Dried product is obtained through a series of post-harvest processing stages [52, 55]. We recorded prices for large- and small-sized individuals for each species, where given, and used only the prices for large-sized individuals in Figures and analyses as a means to improve standardisation of data among respondents. Questionnaires in New Caledonia did not include prices of fresh and dried sea cucumbers, and dried sea cucumbers were rarely sold by fishers in Tonga. Hence, analyses of prices for fresh sea cucumbers excluded New Caledonia, and analyses of prices for dried sea cucumbers included data for Fiji and Kiribati only. Sale prices of fresh and dried sea cucumbers were the current prices at the time of data collection. At the end of interviews, fishers were also encouraged to volunteer any additional information or ask questions.

### Statistical analyses

Linear mixed model (LMM) or generalised linear mixed-model (GLMM) analyses were undertaken for sale prices of dried bêche-de-mer and fresh sea cucumbers, fisher satisfaction and livelihood diversity. Analyses comprised of a random model specification (with random factors) and a fixed model specification (with fixed terms). Three survey design factors, considered as random (‘unallocated’) factors, were: *Country* (*C*), *Location* (*L*) (nested within Countries) and *Village* (*V*) (nested within Locations); further, fishers (*Fisher (F))* were nested within villages. *Surveyor* (*S*) effects were also included in the random model specification. Various covariates from the questionnaires were included in the selection process within fixed model specifications for each response variable (S1 Table), and replicates (fishers) with missing data were excluded from the respective analysis. Diagnostic tools such as Normal Q-Q plots and the residual versus predicted values were used to verify that test assumptions were met.

Some species of sea cucumber were collected seldom by fishers, so analyses of sale prices focussed on the 15 most commonly (overall) caught species. Price data for Fiji and Tonga were converted from FJD and TOP to AUD using international exchange rates at the midpoint of each survey period. Prices for fresh and dried product were log-transformed, which satisfied assumptions of the analysis. Log-transformed prices for dried and fresh product were considered as approximately Gaussian, hence we used a LMM. The model accommodated the nested sampling design and the multiple responses from individual interviews (equivalent to *F*) for data on sale prices. These two features are similar to properties considered by Brien and Demétrio [56] for the analysis of longitudinal data. This approach identifies terms with dependent observations for each fisher, in our case the multiple responses of prices for different species of sea cucumbers, indexed by the fixed factor *Species (Sp)*. The models also retain the principle to include, at least, all terms associated with the random sampling regime. The baseline LMM for the (log) price responses included *Sp* and all interactions of *Sp* with design factors. A saturated model, constructed from this baseline model, included all covariate main effects and covariate interaction effects with *Sp* as fixed and random effects respectively (S1 Table). For these analyses of prices for fresh and dried product, we constructed and included in the models a composite index of prices received by each fisher for each species relative to the country average price for that species (S1 Table).

Livelihood diversity (number income-generating activities) of each fisher is a positive integer, so we used a GLMM with a log-link and Poisson distribution. For data on fisher satisfaction, we conducted three separate analyses to test whether any of the random or fixed factors distinguished the following binary comparisons: (a) any satisfied versus any dissatisfied, (b) satisfied versus very satisfied, and (c) dissatisfied versus very dissatisfied. Use of these derived binary responses overcomes the asymmetry of the measurement scale. The three contrasts of fisher satisfaction were therefore analysed using a GLMM with a binomial distribution and logit link.

To obtain a final model for each response, we used a backward elimination algorithm that respected the marginality of the model terms. The threshold for removing a term was determined from *p*-values for each set of tests, hereafter referred to as families. These were compared to a Family-Wise Error Rate (FWER) threshold [57], calculated as *α*/*m*, where *α* is 0.05 and *m* is the number of tests (terms) within a given family. Sequentially-dropped fixed factors corresponded to the largest *p*-value in each family that was greater than the FWER specific to that family. This approach ensured conservative testing of the model terms to reduce the probability of Type I errors. Terms in the random model specification associated with the survey design or multiple observations factor (*Sp)* were not tested, as advocated by Bailey [58] and Brien and Demétrio [56]. Deviance and Wald tests were used for elimination of fisher livelihood diversity and satisfaction covariates respectively. Likelihood ratio tests (for the random interaction terms) followed by Wald tests (for fixed terms) were used to reduce the models for fresh and dried prices. All analyses were undertaken using the R [59] package, ASReml-R [60], where all predicted values from the fitted models were back-transformed for interpretation.

## Results

The largest sample of interviews was obtained in Fiji, and lowest in New Caledonia, for reasons mentioned earlier (Table 1). Proportionally more women were interviewed in Fiji than in the other countries. Our data show that we sampled across a wide range of fishing modes, ages and genders of fishers [41]. Apart from a bias towards gender inclusiveness, our data can be considered representative of all types of fishers collecting sea cucumber and not just regular fishers, since fishers collecting sea cucumbers occasionally (i.e. 2 or fewer days per week) represented 35%, 12%, 17% and 46% of respondents in Fiji, Kiribati, Tonga and New Caledonia, respectively. Over half of the fishers in each country fished other marine animals than sea cucumbers. Difficulty with selling sea cucumbers or bêche-de-mer was most frequent in Kiribati and least frequent in New Caledonia.

**Table 1:** Replication of socio-economic surveys, diversification of resources fished and difficulty in selling the catch in each country.

Location	Number of respondents (*n*)	Proportion of women (%)	Proportion fishing other resources (%)	Proportion finding difficulties selling sea cucumbers (%)
Fiji	235	26	70	51
Kiribati	84	1	51	73
Tonga	131	19	79	45
New Caledonia	26	15	81	4

### Subsistence consumption of sea cucumbers

**Fig 2:**
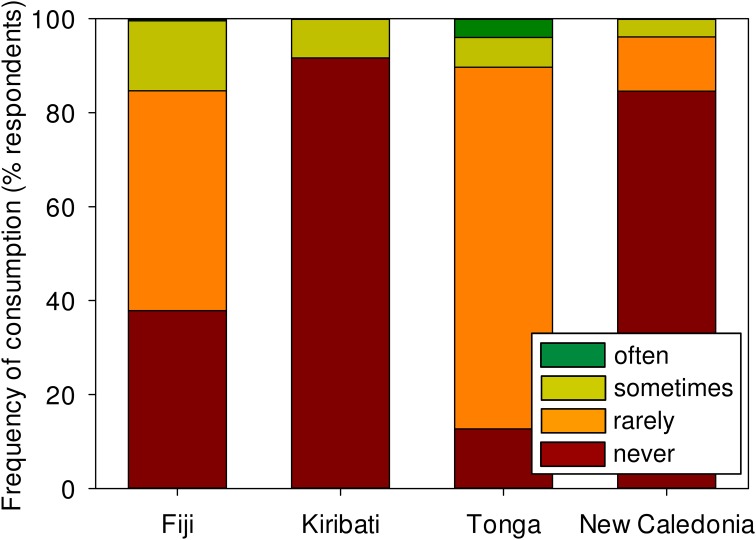
Frequency of subsistence consumption of sea cucumbers by fishers. Stacked bars represent the frequency of consumption of sea cucumbers by fishers in Fiji, Kiribati, Tonga and New Caledonia. The suggested guide to fishers for consumption frequency was: often = once or more per week, sometimes = around once per month, rarely = one or a few times per year, never = never.

Consumption of sea cucumbers by fishers in the four study countries was generally uncommon (Fig 2); fewer than 15% of fishers in each country ate sea cucumbers “sometimes” or “often”. Tongans who ate them would do so on Sundays or festive occasions and, based on additional responses, golden sandfish (*Holothuria lessoni*) was favoured but occasionally some other species were eaten, including brown sandfish (*Bohadschia vitiensis*), chalkfish (*B*. *marmorata*), dragonfish (*S*. *monotuberculatus* and *S*. *horrens*), curryfish (*Stichopus herrmanni*), black teatfish (*H*. *whitmaei*) and white threadsfish (*H*. *leucospilota*) organs. Even still, just 4% of Tongan fishers ate sea cucumbers often. In Fiji, sandfish (*H*. *scabra*) is the species commonly consumed, likewise mostly on Sundays, but a couple other species (e.g. black teatfish) are very occasionally eaten. In Kiribati and New Caledonia, 92% and 85% of fishers, respectively, had never eaten sea cucumbers, either because they considered it disgusting or did not know how to prepare it for consumption.

### Sale of sea cucumbers

#### Fresh product

**Fig 3:**
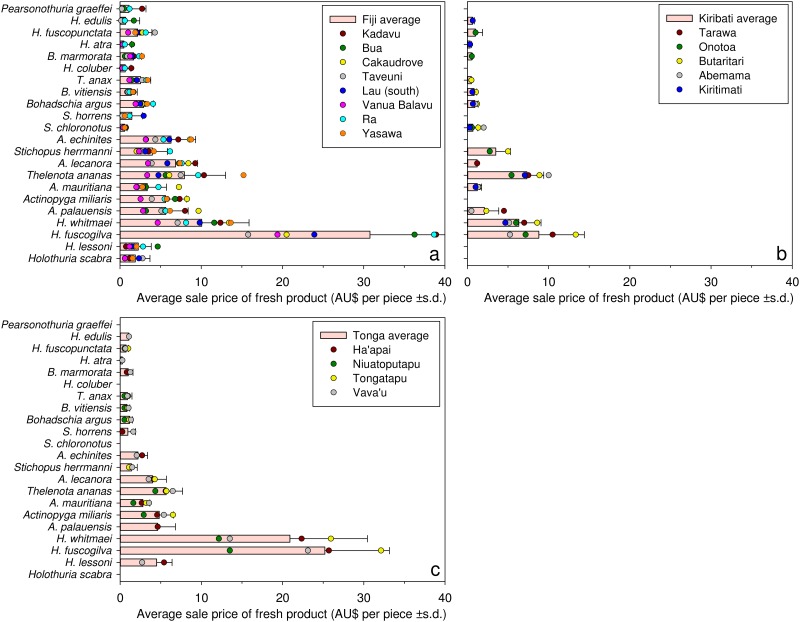
Sale prices from fishers to buyers for fresh sea cucumbers. Bars are country-wide average sale prices of whole fresh (unprocessed) large-sized individuals, of 22 species of sea cucumbers in Fiji (top left), Kiribati (top right) and Tonga (bottom left). Coloured dots are average prices in locations (see inset legends) within each country. Sale is from fishers to exporters or their agents. Average international conversion rates over the survey periods were used to convert data on prices in Fiji (FJD to AUD = 0.58) and Tonga (TOP to AUD = 0.54); the currency in Kiribati is AUD. Comparable data were not collected in surveys with fishers in New Caledonia. Species are arranged in order of descending economic value from the bottom to the top of graphs, according maximum prices in Chinese markets (Purcell 2014).

Of the 22 species on the questionnaires, fishers in Fiji, Tonga, and Kiribati sold 22, 18 and 15 species, respectively, as fresh (unprocessed) product (Fig 3), and some other species were also sold [41]. The raw data indicated higher selling prices for fresh (raw) sea cucumbers in Fiji than in Kiribati or Tonga. White teatfish (*Holothuria fuscogilva*) provided fishers with the highest income per piece, with sample averages ranging from AU$31 (±16 s.d.) in Fiji, to AU$25 (±8 s.d.) in Tonga and AU$9 (±6 s.d.) in Kiribati. Across all respondents, black teatfish was the second-highest value, averaging AU$14 piece^-1^. The lowest-value species was lollyfish (*H*. *atra*), averaging AU$0.45 piece^-1^. Other species providing on average (overall) less than AU$1 piece^-1^ in the fresh form for large specimens were snakefish (*H*. *coluber*), pinkfish (*H*. *edulis*) and greenfish (*Stichopus chloronotus*). Sandfish were sold in several locations in Fiji, and golden sandfish were sold in two locations in Tonga, despite national export bans on those species.

The species-x-gender interaction was statistically significant (*p* < 0.001) in the fitted model for fresh sea cucumbers, so the main effect of gender was retained in the fixed model specification. Geographic clustering of sale prices was evident, whereby considerable variation occurred among countries (13%) and to a lesser extent among locations (10%) and villages (10%). The species-x-country interaction also accounted for a large proportion of total variation (25%); i.e. differences in prices among species were not consistent among countries. For example, the predicted prices were higher for prickly redfish (*Thelenota ananas*) and curryfish in Kiribati than Tonga but not for stonefish (*A*. *lecanora*) or chalkfish.

The gender-x-species interaction accounted for 5% of the total variation in the model; i.e. gender differences in prices for fresh product were species-specific. For example, the predicted prices offered to women for black teatfish in Tonga were less than half of those offered to men. Tongan women, however, received 7% more than men for lollyfish. In Fiji, estimated prices that women received for white teatfish and black teatfish were no more than 60% of that offered to men but gender differences in prices were <20% for curryfish and greenfish. For any given species sold, men were estimated to receive 1.3 times more for their fresh catch than women.

#### Dried product

**Fig 4:**
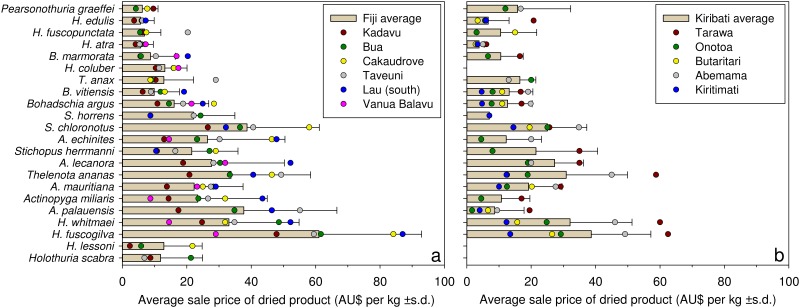
Sale prices from fishers to buyers for dried (fully processed) sea cucumbers. Bars are the country-wide average sale prices, by the kg of dried large-sized individuals, of 22 species of sea cucumbers in Fiji (left) and Kiribati (right). Coloured dots are average prices in locations (see inset legends) within each country. Fishers in Tonga were not processing sea cucumbers and selling the dried products at the time of the surveys. Average international conversion rates over the survey periods were used to convert data on prices in Fiji (FJD to AUD = 0.58) and Tonga (TOP to AUD = 0.54); the currency in Kiribati is AUD. Ra Province and Yasawa Group are not included in Fiji (left) because none of the fishers reported selling dried products. Data were not collected from fishers in New Caledonia. Species are arranged in order of descending economic value from the bottom to the top of graphs, according maximum prices in Chinese markets (Purcell 2014).

Prices for bêche-de-mer were obtained for 22 species in Fiji and 19 species in Kiribati (Fig 4). In both countries, white teatfish was the most valuable species as bêche-de-mer, commanding an average sale price of AU$61 kg^-1^ (± 32 s.d.) in Fiji and AU$39 kg^-1^ (± 19 s.d.) in Kiribati from the fishers sampled. High prices of dried greenfish per kg, despite low prices per piece in the fresh form, is explained by their small size and high proportionate weight loss when processed to bêche-de-mer.

Snakefish, golden sandfish and sandfish were not reported to be sold anywhere in Kiribati. At the time of the study, Tongan fishers were prohibited by national fisheries regulations from doing postharvest processing of sea cucumbers and so could only sell raw product to professional processors/exporters who did the postharvest processing for export. None of the fishers interviewed in Ra province and Yasawa group in Fiji reported selling bêche-de-mer. Absence of data for some locations for certain species was due to buyer preference or geographic distributions of species. For example, i-Kiribati fishers at some locations claimed to have never seen hairy blackfish, deepwater redfish (*A*. *echinites*) or amberfish (*T*. *anax*), but they see snakefish while fishing yet do not harvest it because it is not accepted by buyers.

The random model specification reduced to include only the design factors and their interaction with species, while the fixed effects model reduced to include only species. Variation among fishers explained the greatest proportion of variance (47%) among the random model terms. Variance estimates also indicate some clustering of responses at the level of locations (15%). The species-x-village interaction also explained 7% of the total variation in the random model specification: i.e. there was some variation in species-specific prices depending on the village concerned. The remaining terms explained less than 3% of the total variation in the random model, excluding the residual.

The analysis found higher sale prices for bêche-de-mer in Kiribati than Fiji for 9 of the 15 species. However, of the 3 predicted highest grossing species (white teatfish, black teatfish and greenfish), Fijian fishers were estimated to receive approximately $5–6 more per kg than i-Kiribati fishers for the white teatfish and greenfish species.

### Satisfaction with sale of sea cucumbers

**Fig 5:**
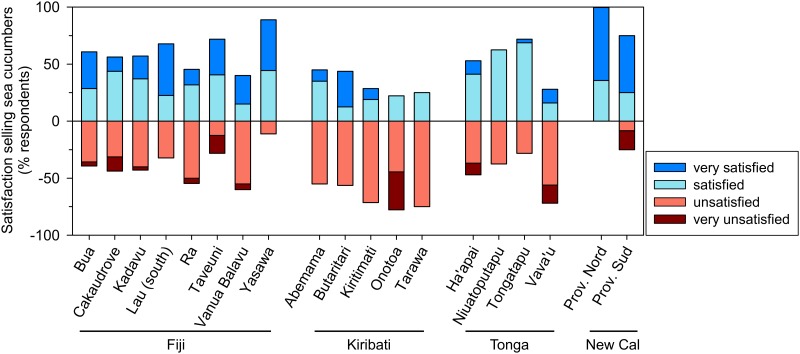
Fishers’ satisfaction with income from fishing and selling sea cucumbers. Fishers were asked how satisfied they were income they gained from fishing and selling sea cucumbers, and to respond according to one of four categories (see legend). Bars represent the proportion of fishers in each location who replied according to the satisfaction categories.

A majority of study locations had a considerable proportion of fishers who were dissatisfied with income from fishing and selling sea cucumbers (Fig 5). Overall, 46% of fishers were dissatisfied or very dissatisfied, and only 19% were very satisfied. Dissatisfied fishers often gave emotive responses, showing they were clearly disgruntled with prices or income from the fishery.

The composite index (*I*) of sale prices was statistically non-significant in the models tested; i.e. the relative price received by fishers for their sea cucumbers did not significantly affect their satisfaction. With New Caledonia data included for subsequent modelling, the logistic regression of satisfied versus unsatisfied fishers found that satisfaction varied greatest among countries (estimated variance = 0.301), and to a lesser extent among locations within countries (estimated variance = 0.196) and there was no evidence of variation in satisfaction among villages. There were more satisfied fishers with one or two livelihood activities and a near even split of satisfied and unsatisfied fishers with >2 income streams but, overall, livelihood diversity of fishers did not significantly affect their satisfaction. Of fishers surveyed in Kiribati, there was more dissatisfaction than satisfaction with selling in each of the five locations (Fig 5), which was reflected in the results. Minor correlations were detected between fishers interviewed by the same surveyor; i.e. fisher satisfaction depended somewhat on who asked the question. Overall, the odds of a male being satisfied with their income from fishing and selling sea cucumbers relative to a female was 0.39; i.e. satisfaction was estimated to be less likely in men. Further, fishers who could easily sell their catch were 3.39 times more likely to be satisfied than fishers who reported difficulty selling their catch due to transport problems, product unwanted by buyers due to surplus, and other issues, with the exception of limited number of buyers.

The contrasts of satisfied versus very satisfied fishers found no model terms to be statistically significant. The second contrast found that age was a significant factor affecting the degree of dissatisfaction. When other terms were held constant, the log-odds of a fisher being very dissatisfied compared to [moderately] dissatisfied increases by 0.052 (5%) with each year increase in age; i.e. dissatisfied fishers become increasingly disgruntled as they become older.

### Income sources and fall-back livelihood options

**Fig 6:**
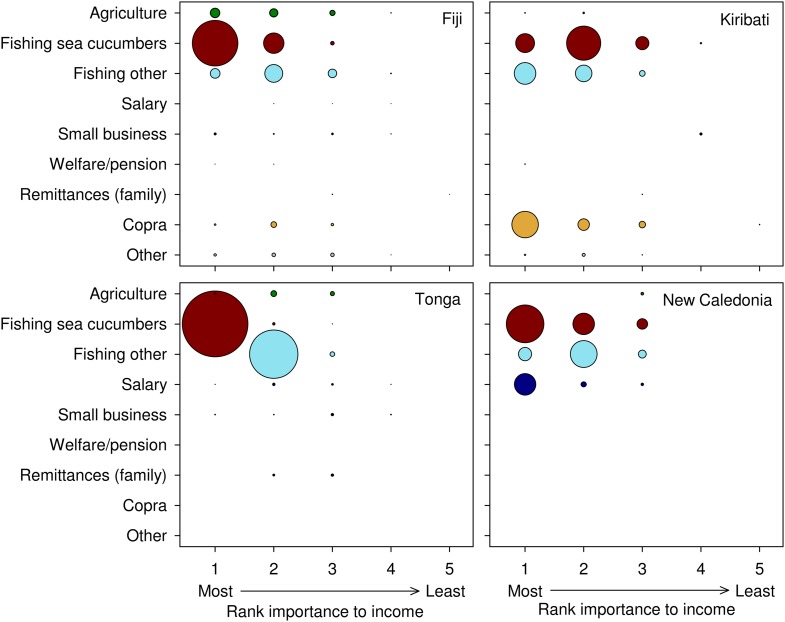
Importance of livelihood activities of fishers. Bubble diameter is scaled to the proportion of fishers relying on each livelihood activity for their most important to least important source of income at the time of the interview. The activity they get most income from was ranked 1, the activity providing the second-most amount of income was ranked 2, and so on. Some fishers only had one or two income sources, while some others had four or five.

In Fiji, Tonga and New Caledonia, sale of sea cucumbers was the primary source of income for a majority of fishers who collected them at the time of our interviews (Fig 6). In particular, sea cucumbers were a primary income source for 95% of Tongan fishers who collected them. In contrast, a greater proportion of i-Kiribati fishers (37%) made most of their income through production of copra (cutting and drying coconuts), the price of which is subsidised by the Kiribati government, than by harvesting and selling sea cucumbers (26%) or other marine resources (30%). However, sea cucumbers were the second-most important income source for 49% of i-Kiribati fishers (Fig 6). Copra was a source of income for few Fijian fishers and no Tongan or New Caledonian fishers. Of all livelihood options, fishing and sale of other marine resources was the next most common income stream for the fishers sampled in Fiji, Tonga and New Caledonia. Salaried income was the next primary income source for fishers in New Caledonia only.

Overall, sea cucumbers were a sole income source for 10% of fishers. The model for livelihood diversity was fully reduced to the null (or intercept) model. That is, the number of livelihood activities of fishers was not found to be significantly affected by gender, age, experience, difficulty selling their catch or by whether a fisher used SCUBA gear. The expected livelihood diversity of fishers in each of the four countries ranged from 2.2–2.5 between locations. Variation in livelihood diversity was only detected at the location level, where livelihood diversity for the remaining blocking terms remained constant. Across all surveys, 64% of fishers had just one or two livelihood activities, and only 4% of fishers had more than three livelihood activities.

**Fig 7:**
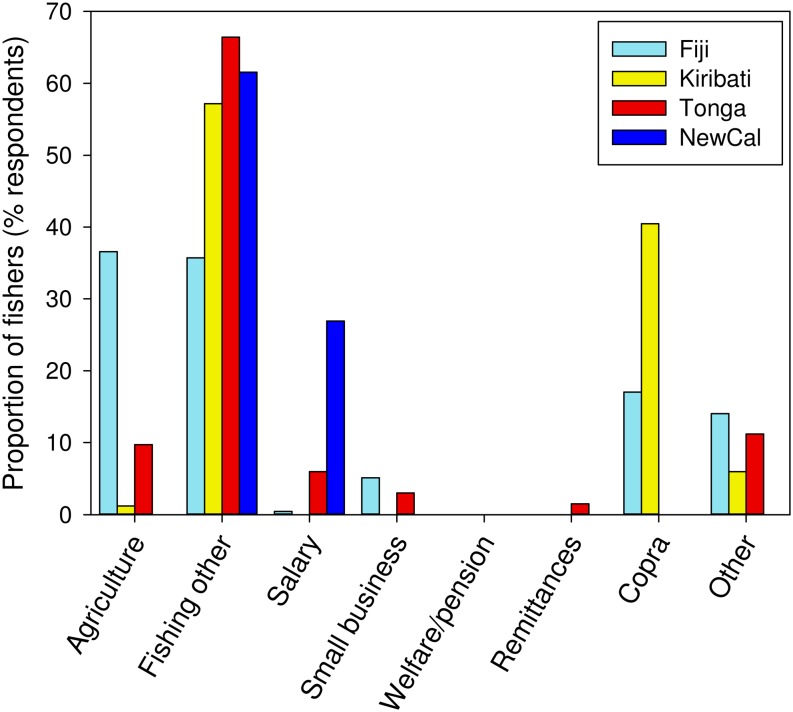
Fall-back livelihood options of fishers. Bars represent the proportion of respondents stating one of eight livelihood activities as a primary fall-back for earning a majority of their income if they could no longer harvest and sell sea cucumbers. Some respondents gave two livelihood activities as fall-back livelihood options, in which case both data responses were included. We recorded copra (collecting, cutting, drying and selling coconut flesh) separate to agriculture because it is not necessarily from planted coconut palms and is not a food crop. ‘Other’ could be livelihood activities including mat weaving, aquarium fish collecting, and other artisanal craft making.

Fall-back livelihood options are activities that fishers would pursue for gaining most of their income if they could no longer collect and sell sea cucumbers. Fishing and selling other marine resources was the most common fall-back livelihood option among countries, except in Fiji, where marginally more fishers would fall back to agriculture (Fig 7). Fishers sometimes elaborated that the other commercial marine resources would be fish, and sometimes lobster, giant clam, shark fin and turtle, if they could no longer harvest sea cucumbers for sale.

## Discussion

### Subsistence consumption of sea cucumbers

Contrary to expectations that subsistence consumption of sea cucumbers is prevalent among Pacific Islanders [51], we found infrequent consumption among fishers who have greatest access to the resource. Regular subsistence consumption of sea cucumbers is known for a few Pacific Islands, such as Samoa [51]. Our finding of occasional consumption in Tonga reflects greater appreciation among Polynesian countries [51]. However, generally infrequent rates of consumption in all four study countries suggest that sea cucumbers represent unsubstantial sources of protein in diets of Pacific islanders, and so over-exploitation of stocks does not directly impact food security.

Our study underscores geographic variability in low-frequency subsistence consumption of sea cucumbers at the between-country scale. Local consumption was very uncommon in New Caledonia, where the GDP per capita is comparatively high [61] and fishers can access a range of local and imported foods. Higher incidences of subsistence consumption of sea cucumbers in Fiji, which is also predominantly Melanesian, reveals that cultural differences in seafood consumption are explained at a country level rather than a regional level. Income does not seem to be driving these consumption patterns among countries since i-Kiribati fishers, who were probably the poorest of the four countries, also had very low rates of consumption. Rare consumption of sea cucumbers in Kiribati might be surprising considering that i-Kiribati have low nutritional standards and fewer locally-available food items [62], and relatively low GDP per capita [61]. The broader inference is that subsistence consumption of certain coastal resources, such as sea cucumbers, is not necessarily about nutritional needs or poverty but rather about culture.

### Variation in sale prices

Variations in sea cucumber prices offered to fishers can be explained by several factors such as poor post-harvesting method [40], remoteness of locations [63], and exploitation of fishers by middlemen [64–66]. Great variation in prices of dried product at the level of individual fishers is indicative of recent findings that some fishers used poor processing practises [52], resulting in damaged bêche-de-mer that would attract low prices. The finding affirms a need for training and information sources in order to improve their processing techniques. Relatively poor prices for fresh product were common among i-Kiribati fishers, who often had difficulties in selling their catch. This could be caused by weak competition among buyers and difficulty in accessing buyers, which arose as potential underlying causes of low fisher satisfaction (discussed later).

Comparisons of bêche-de-mer prices (current at date of surveys) indicate country-specific preferences by exporters for certain species (S2 and S3 Tables). Probable temporal variation in market prices [55] undermines inferences on such comparisons. Nonetheless, prices for dried bêche-de-mer in Kiribati and Fiji compare relatively closely with prices in Yemen [67], Madagascar [68] and Vietnam [69], but were considerably higher than a range of species from Kenya in 2004 [65] (S2 Table). On the other hand, prices for fresh sea cucumbers were generally many times higher in our Pacific Island study countries than other Indo-Pacific countries (S3 Table). Potential market-price variations notwithstanding, the comparisons highlight the lucrative nature of Pacific Island sea cucumber fisheries and explain why many fishers sell sea cucumbers raw rather than processing their catch themselves and selling as dried product.

Sandfish and golden sandfish attract very high prices in the market place if product size is large [55]. Both species have high conversion ratios of fresh to dried product [70, 71], explaining the mediocre value per piece when sold fresh. Low prices for these species relative to other countries (S2 Table) and their consumer value in China [55] could also be related to the small sizes of specimens due to over-exploitation of the populations [55, 63, 72] and because trade is illegal in Fiji and Tonga. Fishers had little understanding of the real market value of sea cucumbers and could be easily exploited by unscrupulous buyers. This was evidenced from volunteered comments from many fishers that they did not know market prices, and from responses that they had never received published information about sea cucumber processing (and prices) [52].

Gendered variation in prices for fresh sea cucumbers could be attributable to economic exploitation by certain buyers and/or because women probably tend to catch smaller animals than men for any given species. As discussed, women tend to fish in shallower waters and specimen sizes are generally smaller in shallows than in deeper waters for the same species. Women are apparently exploited economically by foreign traders in sea cucumber fisheries further afield, such as in Zanzibar [64] and Kenya [65], and the gendered variation in prices we found could result from buyers offering them lower prices than men for the same species.

Large variation in prices of bêche-de-mer at the scale of locations within countries could be explained in part by differing fishing methods. For example, in Fiji SCUBA is banned at Kadavu, so fishers need to collect in shallower waters where animals are probably smaller on average, whereas fishers in southern Lau Group often use SCUBA so are collecting larger animals and so were getting higher prices. Remoteness of a location to export chains could also dampen prices, such as on Kiritimati island, but was not a consistent determinate of prices.

### Satisfaction of fishing and selling sea cucumbers

Small-scale fisheries are a source of livelihood for millions of fishers worldwide [29] and are vital in poverty alleviation in low-income countries [73, 74]. Nonetheless, small-scale fishers share major concerns about regulatory constraints on fishing, marginal incomes, and a lack of alternative livelihood sources [1, 75, 76]. Ruiz [28] found that fishing is satisfying as an occupation, yet fishers can be disgruntled about the level of earnings, the condition of marine resources and the performance of management institutions. Almost half of the fishers in the present study were dissatisfied or disgruntled with the income earned from fishing and selling sea cucumbers. They have a high investment of their time in the fishery [41] and often have few other viable livelihood options (discussed later).

Examples from Tonga highlight how expectations by fishers can strongly affect satisfaction. On the remote island of Niuatoputapu, fishers harvested sea cucumbers only recently so catches were still relatively high [41], which explains why fishers were generally satisfied with their income. On Tongatapu, fishers might have been generally satisfied because occasional gleaning is commonplace and even modest income was satisfactory because fishing costs are minimal. In contrast, fishers in the Vava’u island group were commonly dissatisfied because they expected a set price for sea cucumbers but were sometimes offered lower prices by the few buyers in periods of over-production.

There are multiple dimensions to wellbeing [77–79] and our wellbeing measure is relatively cursory because of the multidisciplinary scope of the questionnaires. However, we show that discontentment with fishery income can be attributed to supply-chain problems and was especially high in Kiribati where atolls are far from the point of export (Tarawa atoll) and transport is limited. Wellbeing is now advocated as an important consideration for development policy [77, 78]. Exploring fisher wellbeing, as part of fishery diagnosis, offers a more holistic means of assessing the social impacts of change in fisheries [77]. In this context, our study reveals opportunities to improve the psychological (and, no doubt, economic) wellbeing of fishers through programs to improve access of fishers to markets, particularly for remote communities such as those on scattered atolls and islands of the Pacific. Such programs could, for example, involve support for cooperatives to auction dried bêche-de-mer on behalf of fishers, or coordination with island councils to overcome transport constraints.

Satisfaction of income by fishers was largely unrelated to selling prices of their catch, probably because of the little information about true market prices. The greater dissatisfaction of fishers with age is curious, and might be related to increasing pressure with age for basic needs to be met [see 26]. In the Dominican Republic, fishers’ satisfaction with meeting basic needs correlated negatively with their age [28]. In our study, older fishers might have more responsibility in providing their household’s basic needs, and were more dissatisfied than younger fishers when problems arose in gaining income from the fishery. Our findings also suggest that the number of years of fishing experience has little bearing on fishers’ income satisfaction compared to other factors.

### Livelihood diversity and fall-back options

Some fishers (10%) had a sole dependency on sea cucumbers as their only livelihood source, while others had one, two, or a few other livelihood activities even if sea cucumbers were their main income source. The other livelihood activities differed among countries, owing to disparate geographies, demography, histories, economies and legislative frameworks.

Fijian fishers apparently enjoyed greater access to agricultural opportunities than fishers in the other countries, for a variety of reasons including culture, land ownership, soil and rainfall. Tongan and New Caledonian fishers have weaker rights to land than Fijians [47], while i-Kiribati fishers live on atoll cays with shallow, infertile soils, regular droughts [80], and poor access to agricultural markets. Meanwhile, i-Kiribati fishers clearly benefit from national revenue from royalties on tuna fishing that allow the government to subsidise one agricultural commodity that can be reliably produced on atolls—copra. In New Caledonia, salary was a significant primary or secondary income source, likely related to a high level of industrial development and a large mining sector. In Tonga, the majority of sea cucumber fishers also catch fish for sale in villages and local markets. Whereas remittances are considered important for many Pacific islanders [81, 82], our study shows their importance in rural communities is not ubiquitous and that they represent a very infrequent income source for artisanal fishers.

Our data highlighted that fishers in Kiribati, Tonga and New Caledonia would mostly turn to other fisheries as a fall-back livelihood option if they were forced to exit the fishery (could no longer harvest and sell sea cucumbers). Exiting of fishers from a fishery might reduce fishing pressure in light of declining stocks [83, 84], yet our study indicates that a high proportion of small-scale fishers would simply shift to other easily-accessible fisheries. Indeed, fishers in developing countries often have limited livelihood options outside the fisheries sector [83–86]. In many instances, those other fisheries were also vulnerable to overfishing or threatened globally (e.g. giant clams, turtle, sharks). This study reveals an underlying co-dependence among small-scale fishery stocks, via artisanal fishers, because they can easily shift effort to other fisheries if economic returns become marginal (i.e. bioeconomic equilibrium) or if fishing is prohibited or constrained. Hence, regulations preventing or restricting fishing in one fishery can have flow-on effects to another, though this likely depends on ecological and economic contexts. In 2015, Kiribati and Tonga imposed moratoria on their sea cucumber fisheries due to concerns of overfishing, and our findings indicate that fishing pressure would have concomitantly increased on other nearshore fisheries. This phenomenon infers that small-scale fisheries should be managed using a holistic approach in which risks to other economically important stocks (which might otherwise experience reduced fishing pressure when other fisheries are open) are managed concurrently.

## Conclusions

Our study confirms that, with the exception of a few Oceania countries with subsistence fisheries [38], overfishing of sea cucumbers is an issue affecting income security but not directly affecting food security. Prices received by fishers for harvested seafood can vary across geographic and demographic factors, and distance from export centres appears to disadvantage some fishers. Evidence of poor knowledge of the market value of sea cucumbers by many fishers, suggests that national pricing standards could improve equality in small-scale fisheries. High variation in selling prices among fishers for processed products underscores great opportunities to improve economic returns in fisheries through training and information sources on postharvest processing. In tandem with strengthening supply chains, such support should benefit the economic and psychological wellbeing of fishers.

Livelihood diversity differed among countries according to a suite of country-specific idiosyncrasies, so livelihood diversification programs will need to be context-specific even within geographic regions. Frequent reliance by fishers on other, sometimes vulnerable, small-scale stocks for secondary and fall-back income highlights connectivity among artisanal fisheries. Such co-dependence among fisheries necessitates a holistic approach to fisheries management in which decision making considers flow-on effects to other easily harvested resources.

## Supporting Information

S1 Table Statistical models and test statistics. (DOCX)

S2 Table Prices per kilogram in AUD for dried sea cucumbers from different countries. (DOCX)

S3 Table Prices per piece (individual animal) in AUD for fresh sea cucumbers from different countries. (DOCX)

## References

[1] Teh LSL, Teh LCL, Sumaila UR. A global estimate of the number of coral reef fishers. PLoS ONE. 2013; 8(6): e65397 10.1371/journal.pone.0065397 23840327 PMC3686796

[2] Gillett R. Fisheries in the economies of the Pacific island countries and territories. Manila, Philippines: Asian Development Bank; 2009.

[3] Berkes F, Mahon R, McConney P, Pollnac R, Pomeroy R. Managing small-scale fisheries: alternative directions and methods. Ottawa, Canada: International Development Research Centre; 2001.

[4] Bell JD, Johnson JE, Hobday AJ, editors. Vulnerability of tropical Pacific fisheries and aquaculture to climate change. Noumea: Secretariat of the Pacific Community; 2011.

[5] Militz TA, Kinch J, Foale S, Southgate PC. Fish rejections in the marine aquarium trade: an initial case study raises concern for village-based fisheries. PLoS ONE. 2016; 11(3): e0151624 10.1371/journal.pone.0151624 26963259 PMC4786313

[6] Namudu MT, Pickering TD. Rapid survey technique using socio-economic indicators to assess the suitability of Pacific Island rural communities for *Kappaphycus* seaweed farming development. J Appl Phycol. 2006; 18(3–5): 241–9.

[7] Carleton C, Hambrey J, Govan H, Medley P, Kinch J. Effective management of sea cucumber fisheries and the beche-de-mer trade in Melanesia. SPC Fisheries Newsletter. 2013; 140(24–42).

[8] Harper S, Zeller D, Hauzer M, Pauly D, Sumaila UR. Women and fisheries: contribution to food security and local economies. Mar Policy. 2013; 39(1): 56–63.

[9] Lambeth L, Hanchard B, Aslin H, Fay-Sauni L, Tuara P, Des Rochers K, et al An overview of the involvement of women in fisheries activities in Oceania. SPC Women in Fisheries Inf Bull. 2014; 25: 21–33.

[10] Zhao M, Tyzack M, Anderson R, Onoakpovike E. Women as visible and invisible workers in fisheries: A case study of Northern England. Mar Policy. 2013; 37: 69–76.

[11] Fröcklin S, De La Torre-Castro M, Håkansson E, Carlsson A, Magnusson M, Jiddawi NS. Towards improved management of tropical invertebrate fisheries: including time series and gender. PLoS ONE. 2014; 9(3): e91161 10.1371/journal.pone.0091161 24614075 PMC3948745

[12] Weeratunge N, Snyder KA, Sze CP. Gleaner, fisher, trader, processor: understanding gendered employment in fisheries and aquaculture. Fish Fish. 2010; 11(4): 405–20.

[13] Sugiyama S, Staples D, Funge-Smith SJ. Status and potential of fisheries and aquaculture in Asia and the Pacific. Food and Agriculture Organization Regional Office for Asia and the Pacific, Publication 2004/25. 2004.

[14] Christensen AE. Making an island living: continuity and change on Ontong Java, Solomon Islands. Copenhagen: University of Copenhagen; 2010.

[15] Christensen AE. Marine gold and atoll livelihoods: the rise and fall of the bêche-de-mer trade on Ontong Java, Solomon Islands. Nat Resour Forum. 2011; 35: 9–20.

[16] Foale SJ. Sharks, sea slugs and skirmishes: managing marine and agricultural resources on small, overpopulated islands in Milne Bay, PNG. RMAP Working Paper. Canberra: Resource Management in Asia Pacific Program, The Australian National University, 2005 December 2005. Report No. 64

[17] Hair C, Foale S, Kinch J, Yaman L, Southgate PC. Beyond boom, bust and ban: the sandfish (*Holothuria scabra*) fishery in the Tigak Islands, Papua New Guinea. Reg Stud Mar Sci. 2016; 5: 69–79.

[18] Koczberski G, Curry G, Warku J, Kwam C. Village-based marine resource use and rural livelihoods, Kimbe Bay, West New Britain, Papua New Guinea. Brisbane: The Nature Conservancy, USAID, Curtin University, 2006 October 2006. Report No.: TNC Pacific Island Countries Report No 5/06.

[19] Reddy SMW, Groves T, Nagavarapu S. Consequences of a government-controlled agricultural price increase on fishing and the coral reef ecosystem in the Republic of Kiribati. PLoS ONE. 2014; 9(5): e96817 10.1371/journal.pone.0096817 24820734 PMC4018407

[20] Campbell JR. Development, global change and traditional food security in Pacific Island countries. Reg Environ Change. 2015; 15(7): 1313–24.

[21] Browne C. Pacific Island economies. Washington, D.C: International Monetary Fund; 2006.

[22] Coulthard S, Johnson D, McGregor JA. Poverty, sustainability and human wellbeing: a social wellbeing approach to the global fisheries crisis. Global Environ Chang. 2011; 21(2): 453–63.

[23] Coulthard S. More than just access to fish: The pros and cons of fisher participation in a customary marine tenure (Padu) system under pressure. Mar Policy. 2011; 35(3): 405–12.

[24] Pollnac RB, Poggie JJ. Job satisfaction in the fishery in two Southeast Alaskan towns. Hum Organ. 2006; 65(3): 329–39.

[25] Pollnac RB, Pomeroy RS, Harkes IHT. Fishery policy and job satisfaction in three southeast Asian fisheries. Ocean Coast Manage. 2001; 44(7–8): 531–44.

[26] Coulthard S, Paranamana N, Sandaruwan L, Manimohan R, Maya R, Amarasinghe O, et al Exploring wellbeing in fishing communities (South Asia): Methods handbook. UK: University of Northumbria; 2015.

[27] Trimble M, Johnson D. Artisanal fishing as an undesirable way of life? The implications for governance of fishers' wellbeing aspirations in coastal Uruguay and southeastern Brazil. Mar Policy. 2013; 37(1): 37–44.

[28] Ruiz V. Job satisfaction among fishers in the Dominican Republic. Soc Indic Res. 2012; 109(1): 81–94.

[29] FAO. The state of world fisheries and aquaculture: opportunities and challenges. Rome: FAO; 2014.

[30] Gillett R. Fisheries of the Pacific Islands: regional and national information. Bangkok: Food and Agriculture Organization of the United Nations. Regional Office for Asia and the Pacific; 2011 279 p.

[31] Gillett R, Lightfoot C. The contribution of fisheries to the economies of Pacific Island countries. Manila, Philippines: Asian Development Bank; 2001.

[32] Caldwell J, Missingham B, Marck J. The population of Oceania in the second millennium. Canberra: Health Transition Centre, The Australian National University, 2001 26 September 2001. Report No.

[33] Foale SJ, Day RW. Stock assessment of trochus (*Trochus niloticus*) fisheries at West Nggela, Solomon Islands, with notes on management. Fish Res. 1997; 33: 1–16.

[34] Friedman K, Eriksson H, Tardy E, Pakoa K. Management of sea cucumber stocks: patterns of vulnerability and recovery of sea cucumber stocks impacted by fishing. Fish Fish. 2011; 12(1): 75–93.

[35] Friedman K, Kronen M, Pinca S, Magron F, Boblin P, Pakoa K, et al. Papua New Guinea country report: profiles and results from survey work at Andra, Tsoilaunung, Sidea and Panapompom. Noumea: Secretariat of the Pacific Community; 2009. 268 p.

[36] Green A, Lokani P, Atu W, Ramohia P, Thomas P, Almany J. Solomon Islands marine assessment: technical report of survey conducted May 13 to June 17, 2004. Brisbane: The Nature Conservancy, 2006.

[37] Eriksson H, Conand C, Lovatelli A, Muthiga NA, Purcell SW. Governance structures and sustainability in Indian Ocean sea cucumber fisheries. Mar Policy. 2015; 56: 16–22.

[38] Purcell SW, Mercier A, Conand C, Hamel JF, Toral-Granda MV, Lovatelli A, et al Sea cucumber fisheries: global analysis of stocks, management measures and drivers of overfishing. Fish Fish. 2013; 14(1): 34–59.

[39] Pakoa K, Bertram I. Management state of Pacific sea cucumber fisheries. SPC Beche-de-mer Inf Bull. 2013; 33: 49–52.

[40] Purcell SW, Lovatelli A, Pakoa K. Constraints and solutions for managing Pacific Island sea cucumber fisheries with an ecosystem approach. Mar Policy. 2014; 45: 240–50.

[41] Purcell SW, Ngaluafe P, Tamuera KT, Lalavanua W. Trends in small-scale artisanal fishing of sea cucumbers in Oceania. Fish Res. 2016; 183: 99–110.

[42] Kirch PV. On the road of the winds: an archaeological history of the Pacific Islands before European contact. San Francisco: University of California Press; 2000.

[43] Shineberg D. The sandalwood trade in Melanesian economics, 1841–65. J Pac Hist. 1966; 1: 129–46.

[44] Bennett JA. Wealth of the Solomons A history of a Pacific archipelago, 1800–1978. Honolulu: University of Hawaii Press; 1987 529 p.

[45] Conand C. The fishery resources of Pacific Island countries. Pt. 2: Holothurians. FAO Fisheries Technical Paper 272.2. Rome: FAO; 1990. 153 p.

[46] Kinch J, Purcell S, Uthicke S, Friedman K. Papua New Guinea: a hot spot of sea cucumber fisheries in the Western Central Pacific. FAO Fisheries and Aquaculture Technical Paper No. 516. In: Toral-Granda V, Lovatelli A, Vasconcellos M, editors. Sea cucumbers: A global review of fisheries and trade. Rome: FAO; 2008. p. 57–77.

[47] Ward RG. The Pacific Bêche-de-mer trade with special reference to Fiji In: Ward RG, editor. Man in the Pacific Islands. Oxford: Clarendon Press; 1972 p. 91–121.

[48] Choo PS. Population status, fisheries and trade of sea cucumbers in Asia. In: Toral-Granda V, Lovatelli A, Vasconcellos M, editors. Sea cucumbers: A global review of fisheries and trade. Rome: FAO Fisheries and Aquaculture Technical Paper No. 516; 2008. p. 81–118.

[49] Conand C. Population status, fisheries and trade of sea cucumbers in Africa and the Indian Ocean. In: Toral-Granda V, Lovatelli A, Vasconcellos M, editors. Sea cucumbers: A global review of fisheries and trade. Rome: FAO Fisheries and Aquaculture Technical Paper No. 516; 2008. p. 143–93.

[50] Hamel J-F, Mercier A. Population status, fisheries and trade of sea cucumbers in temperate areas of the northern hemisphere. In: Toral-Granda V, Lovatelli A, Vasconcellos M, editors. Sea cucumbers: A global review of fisheries and trade. Rome: FAO Fisheries & Aquaculture Technical Paper No. 516; 2008. p. 257–92.

[51] Kinch J, Purcell S, Uthicke S, Friedman K. Population status, fisheries and trade of sea cucumbers in the Western Central Pacific. In: Toral-Granda V, Lovatelli A, Vasconcellos M, editors. Sea cucumbers: A global review of fisheries and trade. FAO Fisheries and Aquaculture Technical Paper No 516. Rome: FAO; 2008. p. 7–55.

[52] Purcell SW, Ngaluafe P, Aram KT, Lalavanua W. Variation in postharvest processing of sea cucumbers by fishers and commercial processors among three Pacific Island countries. SPC Beche-de-mer Inf Bull. 2016; 36: 58–66.

[53] Purcell SW, Gossuin H, Agudo NS. Status and management of the sea cucumber fishery of La Grande Terre, New Caledonia WorldFish Center Studies and Review 1901. Penang: WorldFish Center; 2009 136 p.

[54] Kleiber D, Harris LM, Vincent ACJ. Gender and small-scale fisheries: a case for counting women and beyond. Fish Fish. 2015; 16(4): 547–62.

[55] Purcell SW. Value, market preferences and trade of beche-de-mer from Pacific Island sea cucumbers. PLoS ONE. 2014; 9(4): e95075 10.1371/journal.pone.0095075 24736374 PMC3988149

[56] Brien CJ, Demétrio CGB. Formulating mixed models for experiments, including longitudinal experiments. J Agr Biol Envir St. 2009; 14(3): 253–80.

[57] Benjamini Y, Hochberg Y. Controlling the false discovery rate: a practical and powerful approach to multiple testing. J Roy Stat Soc Ser B (Stat Method). 1995; 57(1): 289–300.

[58] Bailey RA. Design of Comparative Experiments. Cambridge: Cambridge University Press; 2008.

[59] R-Core-Team. R: a language and environment for statistical computing. Vienna, Austria: R Foundation for Statistical Computing; 2015.

[60] Butler D. asreml: asreml() fits the linear mixed model. R package version 3.0. 2009.

[61] UNDP. Human development report 2015: work for human development. Human development statistical tables: UNDP; 2015 [cited 2015 30-5-16]. http://hdr.undp.org/en/data.

[62] Thomas FR. Self-reliance in Kiribati: contrasting views of agricultural and fisheries production. Geogr J. 2002; 168(2): 163–77.

[63] Pakoa KM, Ngaluafe PV, Lotoahea T, Matoto SV, Bertram I. The status of Tonga’s sea cucumber fishery, including an update on Vava’u and Tongatapu. Noumea, New Caledonia: Secretariat of the Pacific community; 2013 46 p.

[64] Eriksson HB, de la Torre-Castro M, Eklöf J, Jiddawi N. Resource degradation of the sea cucumber fishery in Zanzibar, Tanzania: a need for management reform. Aquat Living Resour. 2010; 23(04): 387–98.

[65] Ochiewo J, de la Torre-Castro M, Muthama C, Munyi F, Nthuta JM. Socio-economic features of sea cucumber fisheries in southern coast of Kenya. Ocean Coast Manage. 2010; 53(4): 192–202.

[66] Ram R. Impacts of harvest and post harvest processing methods on quality and value of beche-de-mer in Fiji Islands. Suva, Fiji: University of the South Pacific; 2008.

[67] Lindsay S, Al-Agwan Z. Sea cucumber fisheries of Yemen: status and recommendations. Jeddah: PERSGA, 2009.

[68] Lavitra T, Rachelle D, Rasolofonirina R, Jangoux M, Eeckhaut I. Processing and marketing of holothurians in the Toliara region, southwestern Madagascar. SPC Beche-de-mer Inf Bull. 2008; 28: 24–33.

[69] del Mar Otero-Villanueva M, Ut VN. Sea cucumber fisheries around Phu Quoc Archipelago: A cross-border issue between South Vietnam and Cambodia. SPC Beche-de-mer Inf Bull. 2007; 25: 32–6.

[70] Purcell SW, Gossuin H, Agudo NS. Conversion of weight and length of sea cucumbers to beche-de-mer: filling gaps for some exploited tropical species. SPC Beche-de-mer Inf Bull. 2009; 29: 3–6.

[71] Skewes T, Smith L, Dennis D, Rawlinson N, Donovan A, Ellis N. Conversion ratios for commercial beche-de-mer species in Torres Strait: Australian Fisheries Management Authority, Torres Strait Research Program, Final Report.; 2004. 20 p.

[72] Pakoa K, Saladrau W, Lalavanua W, Valotu D, Tuinasavusavu I, Sharp M, et al The status of sea cucumber resources and fisheries management in Fiji. Noumea, New Caledonia: Secretariat of the Pacific Community; 2013 49 p.

[73] Allison EH, Ellis F. The livelihoods approach and management of small-scale fisheries. Mar Policy. 2001; 25(5): 377–88.

[74] Béné C, Hersoug B, Allison EH. Not by rent alone: Analysing the pro-poor functions of small-scale fisheries in developing countries. Dev Policy Rev. 2010; 28(3): 325–58.

[75] Cinner JE, Bodin O. Livelihood diversification in tropical coastal communities: a network-based approach to analyzing 'livelihood landscapes'. PloS One. 2010; 5(8): e11999 10.1371/journal.pone.0011999 20711442 PMC2920305

[76] Kittinger JN, Teneva LT, Koike H, Stamoulis KA, Kittinger DS, Oleson KLL, et al From reef to table: social and ecological factors affecting coral reef fisheries, artisanal seafood supply chains, and seafood security. PLoS ONE. 2015; 10(8): e0123856 10.1371/journal.pone.0123856 26244910 PMC4526684

[77] Coulthard S. What does the debate around social wellbeing have to offer sustainable fisheries? Curr Opin Environ Sustainability. 2012; 4(3): 358–63.

[78] Young MAL, Foale S, Bellwood DR. Why do fishers fish? A cross-cultural examination of the motivations for fishing. Mar Policy. 2016; 66: 114–23.

[79] Fabinyi M, Foale S, Macintyre M. Managing inequality or managing stocks? An ethnographic perspective on the governance of small-scale fisheries. Fish Fish. 2015; 16: 471–85.

[80] Catala RLA. Report on the Gilbert Islands: some aspects of human ecology. Washington DC: Atoll Research Bulletin No. 59. Issued by The Pacific Science Board, National Academy of Sciences—National Research Council.; 1957. 187 p.

[81] Connell J. Islands at risk? Environments, economies and contemporary change. Cheltenham: Edward Elgar; 2013 351 p.

[82] Connell J, Brown RPC. Remittances in the Pacific: an overview. Asian Development Bank, 2005 March 2005. Report No.

[83] Muallil RN, Geronimo RC, Cleland D, Cabral RB, Doctor MV, Cruz-Trinidad A, et al Willingness to exit the artisanal fishery as a response to scenarios of declining catch or increasing monetary incentives. Fish Res. 2011; 111(1–2): 74–81.

[84] Purcell SW. Managing sea cucumber fisheries with an ecosystem approach. FAO Fisheries and Aquaculture Technical Paper No. 520. Rome: FAO; 2010.

[85] Cinner JE, Daw T, McClanahan TR. Socioeconomic factors that affect artisanal fishers' readiness to exit a declining fishery. Conserv Biol. 2009; 23(1): 124–30. 10.1111/j.1523-1739.2008.01041.x 18778267

[86] Jentoft S, Chuenpagdee R, Bundy A, Mahon R. Pyramids and roses: Alternative images for the governance of fisheries systems. Mar Policy. 2010; 34(6): 1315–21.

