# Supplementary material for: Multiple Factors Affect Socioeconomics and Wellbeing of Artisanal Sea Cucumber Fishers
Source: PLoS One. 2016 Dec 8;11(12):e0165633. doi: 10.1371/journal.pone.0165633 (PMC5145150; doi:10.1371/journal.pone.0165633)
Supplement: S1 Table — (DOCX) [file pone.0165633.s001.docx]

**Supplementary material S1**

**Statistical models and test statistics**

Table 1a. Summary of the model terms included (🗸) in the saturated model for the analysis of fisher satisfaction, livelihood options and the sale prices of fresh and dried sea cucumbers. Note that ":" denotes the interaction of two terms.

| **Model Terms** | **Number of livelihoods** | | **Fisher satisfaction** | | **Fresh prices** | | **Dried prices** |
| --- | --- | --- | --- | --- | --- | --- | --- |
| FIXED MODEL |  |  | |  | |  | |
| Age | ✓ | ✓ | | ✓ | | ✓ | |
| Gender | ✓ | ✓ | | ✓ | | ✓ | |
| Experience | ✓ | ✓ | | ✓ | | ✓ | |
| Catch | ✓ | ✓ | |  | |  | |
| Days.F | ✓ | ✓ | |  | |  | |
| Time | ✓ | ✓ | |  | |  | |
| Scuba | ✓ | ✓ | |  | |  | |
| LRea, ORea, TRea & URea | ✓ | ✓ | | ✓ | | ✓ | |
| CPUE |  | ✓ | |  | |  | |
| Index |  | ✓ | |  | |  | |
| LiveOpt |  | ✓ | |  | |  | |
| BAbs, Cabs, FAbs, OAbs, RAbs & WAbs |  | ✓ | |  | |  | |
| Species |  |  | | ✓ | | ✓ | |
| ProcTime |  |  | |  | | ✓ | |
| DTec |  |  | |  | | ✓ | |
| CBoi, FBoi, OBoi, SBoi & YBoi |  |  | |  | | ✓ | |
| Smoked |  |  | |  | | ✓ | |
| Sp.FM:Salt |  |  | |  | | ✓ | |
| Sp.FM:ACut, Sp.FM:DCut, Sp.FM:MCut & Sp.FM:VCut |  |  | |  | | ✓ | |
| Sp.FM:Boil.F, Sp.Fm:Boil.S & Sp.FM:Boil.T |  |  | |  | | ✓ | |
| RANDOM MODEL |  |  | |  | |  | |
| Country | ✓ | ✓ | | ✓ | | ✓ | |
| Region | ✓ | ✓ | | ✓ | | ✓ | |
| Location | ✓ | ✓ | | ✓ | | ✓ | |
| Surveyor | ✓ | ✓ | | ✓ | | ✓ | |
| Fisher |  |  | | ✓ | | ✓ | |
| Species:$(x_{1}+x_{2}+\ldots+x_{k})$ |  |  | | ✓ | | ✓ | |
| Sp.F: $(x_{k+1}+x_{k+2}+\ldots+x_{n})$ |  |  | |  | | ✓ | |

**Table 1b.** **Results for the selling price of dried sea cucumbers.** Boundary terms (with a variance ~ 0), were dropped from the model immediately and then the random and fixed models were reduced via the process of backward elimination. REML log-likelihood ratio tests were used to reduce the random model followed by Wald tests for the fixed effects.

| **Terms Removed** | ***p*-value** | **REML Log-Likelihood** |
| --- | --- | --- |
| **Random Terms** |  |  |
| **Boundary Terms**: Species:(Gender + ORea + TRea + URea + DTec + OBoi + SBoi + YBoi) + Sp.F(Salt + Boil.F + Boil.S + Sp.F:DCut + Sp.F:VCut) | - | 9.89 |
| Species:Age | 0.81 | 9.87 |
| Species:LRea | 0.64 | 9.76 |
| Species:ACut | 0.64 | 9.65 |
| Species:ProcTime | 0.23 | 8.93 |
| Sp.F:MCut | 0.25 | 8.26 |
| Species:Smoked | 0.19 | 7.40 |
| Species:Experience | 0.19 | 6.53 |
| Species:CBoi | 0.11 | 5.27 |
| Species:FBoi | 0.06 | 3.47 |
| **Fixed Terms** |  |  |
| ProcTime | 0.89 | 6.36 |
| Sp:FM:Salt | 0.88 | 10.27 |
| TRea | 0.88 | 11.64 |
| SBoi | 0.86 | 13.57 |
| CBoi | 0.73 | 15.35 |
| Experience | 0.71 | 20.13 |
| Sp.FM:Boil.S | 0.62 | 26.16 |
| FBoi | 0.60 | 27.88 |
| YBoi | 0.58 | 29.73 |
| Age | 0.52 | 38.20 |
| Smoked | 0.40 | 29.61 |
| Sp.FM:VCut | 0.50 | 31.76 |
| Sp.FM:DCut | 0.40 | 33.93 |
| Sp.FM:MCut | 0.42 | 36.35 |
| ORea | 0.35 | 37.62 |
| Gender | 0.30 | 38.97 |
| ACut | 0.25 | 42.27 |
| LRea | 0.16 | 43.50 |
| DTec | 0.07 | 42.61 |
| URea | 0.11 | 43.64 |
| OBoi | 0.14 | 44.36 |
| Sp.FM:Boil.F | 0.05 | 49.07 |

**Table 1c.** **Results for the sale prices of fresh (unprocessed) sea cucumbers.** Boundary terms (with a variance ~ 0), were dropped from the model immediately and then the random and fixed models were reduced via the process of backward elimination. REML log-likelihood ratio tests were used to reduce the random model followed by Wald tests for the fixed effects.

| **Terms Removed** | ***p*-value** | **REML Log-Likelihood** |
| --- | --- | --- |
| **Random Terms** |  |  |
| **Boundary Terms**: Species:(Age + Experience + ORea + TRea + URea) | - | 770.40 |
| Species:LRea | 0.33 | 770.30 |
| **Fixed Terms** |  |  |
| LRea | 0.86 | 772.82 |
| TRea | 0.74 | 775.68 |
| URea | 0.63 | 777.62 |
| Age | 0.55 | 789.96 |
| ORea | 0.21 | 795.16 |
| Experience | 0.03 | 799.25 |

**Table 1d.** **Results of generalized linear mixed model analysis of livelihood diversity of fishers.** Analysis of deviance was used to reduce the fixed model for livelihood diversity via backward elimination. The resulting deviance for the model is presented for the subsequent removal of the relevant term.

| **Terms Removed** | ***p*-value** | **Deviance** |
| --- | --- | --- |
| **Saturated model** |  | -94.08 |
| **Terms with larger deviance than saturated model** |  |  |
| Gender | - | -94.01 |
| Scuba | - | -93.99 |
| LRea | - | -93.99 |
| **Backward elimination of remaining fixed terms** |  |  |
| TRea | 1 | -94.08 |
| URea | 1 | -94.19 |
| ORea | - | -93.48 |
| Time | 1 | -93.54 |
| Experience | 1 | -93.82 |
| Days.F | 1 | -94.27 |
| Age | 1 | -95.19 |
| Catch | 1 | -97.52 |

**Table 1e. Results of generalized linear mixed model analysis of fisher satisfaction with income from fishing and selling sea cucumbers. Contrast 1: any satisfied (+2 and +1) versus any dissatisfied (-1 and -2).** Wald tests were used to reduce the fixed model via backward elimination for contrast 1: any satisfied (+2 and +1) versus any dissatisfied (-1 and -2) fishers. The resulting deviance for the removal of the relevant term from the model is also presented.

| **Terms Removed** | ***p*-value** | **Deviance** |
| --- | --- | --- |
| **Fixed Terms** |  |  |
| FAbs | 0.98 | 445.22 |
| ORea | 0.82 | 445.00 |
| URea | 0.76 | 445.40 |
| CAbs | 0.69 | 445.95 |
| WAbs | 0.65 | 445.49 |
| Experience | 0.65 | 446.38 |
| LiveOpt | 0.53 | 452.01 |
| RAbs | 0.45 | 452.73 |
| Days.F | 0.43 | 454.21 |
| Time | 0.35 | 455.63 |
| Catch | 0.24 | 455.67 |
| Index | 0.22 | 517.27 |
| Age | 0.15 | 526.29 |
| CPUE | 0.09 | 530.07 |
| OAbs | 0.06 | 533.48 |
| TRea | 0.03 | 535.89 |
| BAbs | 0.03 | 543.49 |

**Table 1f.** **Results of generalized linear mixed model analysis of fisher satisfaction with income from fishing and selling sea cucumbers. Contrast 2: satisfied (+1) versus very satisfied (+2).** Wald tests were used to reduce the fixed model via backward elimination for contrast 2: satisfied (+1) versus very satisfied (+2) fishers. The resulting deviance for the removal of the relevant term from the model is also presented.

| **Terms Removed** | ***p*-value** | **Deviance** |
| --- | --- | --- |
| **Fixed Terms** |  |  |
| WAbs | 0.81 | 220.47 |
| Experience | 0.78 | 221.13 |
| ORea | 0.79 | 222.47 |
| Gender | 0.74 | 222.53 |
| URea | 0.72 | 222.36 |
| Catch | 0.64 | 222.07 |
| LiveOpt | 0.61 | 229.99 |
| Index | 0.48 | 296.05 |
| CAbs | 0.66 | 298.21 |
| Days.F | 0.64 | 299.82 |
| RAbs | 0.57 | 300.42 |
| OAbs | 0.46 | 300.76 |
| Time | 0.33 | 302.40 |
| TRea | 0.30 | 305.67 |
| Age | 0.21 | 306.09 |
| LRea | 0.21 | 304.65 |
| BAbs | 0.13 | 305.33 |
| FAbs | 0.05 | 307.70 |

**Table 1g.** **Results of generalized linear mixed model analysis of fisher satisfaction with income from fishing and selling sea cucumbers. Contrast 3: dissatisfied (-1) versus very dissatisfied (-2).** Wald tests were used to reduce the fixed model via backward elimination for contrast 3: dissatisfied (-1) versus very dissatisfied (-2) fishers. The resulting deviance for the removal of the relevant term from the model is also presented.

| **Terms Removed** | ***p*-value** | **Deviance** |
| --- | --- | --- |
| **Fixed Terms** |  |  |
| Gender | 0.97 | 78.28 |
| FAbs | 0.90 | 79.56 |
| URea | 0.84 | 81.65 |
| Days.F | 0.90 | 82.40 |
| CPUE | 0.84 | 83.27 |
| Time | 0.74 | 85.65 |
| Experience | 0.69 | 85.97 |
| Index | 0.68 | 104.15 |
| TRea | 0.45 | 106.33 |
| RAbs | 0.44 | 106.38 |
| OAbs | 0.47 | 108.10 |
| CAbs | 0.28 | 107.91 |
| Catch | 0.32 | 107.90 |
| LiveOpt | 0.18 | 112.35 |
| BAbs | 0.16 | 116.85 |
| LRea | 0.18 | 129.08 |
| ORea | 0.95 | 129.66 |
| WAbs | 0.06 | 136.09 |

**Definition of model terms:**

**Age:** Covariate coding a fisher’s age

**Gender:** Factor with two levels denoting male and female fishers respectively

**Experience:** Covariate coding number of years fishing experience

**LRea:** Factor with three levels coding fishers who find it difficult to sell their catch due to transport issues (TRea), unwanted by the buyer (Urea) and/or other reasons (ORea), fishers who find it difficult to sell their catch due to limited buyers (LRea) and fishers who don’t find it difficult to sell their catch

**ORea:** Factor with three levels coding fishers who find it difficult to sell their catch due to LRea, TRea and/or URea, fishers who find it difficult to sell their catch due to other reasons (ORea) and fishers who don’t find it difficult to sell their catch

**TRea:** Factor with three levels coding fishers who find it difficult to sell their catch due to LRea, ORea and/or URea, fishers who find it difficult to sell their catch due to transport issues (TRea) and fishers who don’t find it difficult to sell their catch

**URea:** Factor with three levels coding fishers who find it difficult to sell their catch due to LRea, ORea and/or TRea, fishers who find it difficult to sell their catch due to the product being unwanted by the buyer (URea) and fishers who don’t find it difficult to sell their catch

**Catch:** Binary factor coding if they only catch sea cucumbers whilst fishing

**Days.F:** Covariate coding average number of days spent fishing/week

**Time:** Covariate coding hours/day spent fishing

**Scuba:** Binary factor coding whether or not SCUBA equipment is used whilst fishing sea cucumbers

**CPUE:** Covariate coding number of pieces caught/hour

**Index:** The price a fisher reported for any given species in a particular state (fresh or dried) was centred by the country average for that species in the appropriate state. For fisher $i$in country $j$, the centred price for each relevant species $k$is defined as

$P_{i,j,k}=(x_{i,j,k}-\bar{x}_{j,k})/\bar{x}_{j,k}$, where $\bar{x}_{j,k}$denotes the average price for species $k$in country $j$. Therefore, the price index for each fisher $i$in country $j$is given by

$I_{i,j}=\sum_{k\in s_{i}} (x_{i,j,k}-\bar{x}_{j,k})/\bar{x}_{j,k}$, where $s_{i}$denotes the set of all species sold by fisher $i$in country $j$

**LiveOpt:** Covariate corresponding to the total number of livelihood options (income sources) of each fisher.

**BAbs, CAbs, FAbs, OAbs, RAbs & WAbs:** Factor with three levels defined similarly to the reasons for difficulty selling catch. First level corresponds to those who are absent from fishing but not for the reason indicated by the factor name, next is those who are absent due to that defined by the factor name that corresponds to bad weather (BAbs), ceremonial/cultural obligations (Cabs), closed fishery (FAbs), rest/vacation (RAbs), other work commitments (WAbs) and/or other reasons (OAbs). The third level corresponds to fishers that are not absent from fishing

**Species:** Factor with 15 levels each corresponding to the name of a different species

**ProcTime:** Covariate corresponding to the hours taken to process the catch

**DTec:** Factor with three levels coding those who use fire as a drying technique, artisanal oven or no drying technique respectively

**CBoi, FBoi, OBoi, SBoi & YBoi:** Factor with three levels defined similarly to the reasons for difficulty selling catch and absence. First level corresponds to the person who boils the catch not identified in the factor name, next is the person who boils the catch denoted by children (CBoi), other family members (FBoi), non-relatives (OBoi), spouse (SBoi) and the fisher being interviewed (YBoi). The third level corresponds to fishers that do not boil their catch

**Smoked:** A binary factor that codes if the catch is smoked or not

**Sp.FM & Sp.F:** Factors for the fixed and random models respectively that correspond to the species for which cut, boil and salt information was collected on to ensure estimates are only generated for those species that information has been collected on

**Salt:** Covariate coding days spent salting the catch

**ACut, DCut, MCut & VCut:** Factor with three levels defined similarly to the reasons for difficulty selling catch, absence and boilers. First level corresponds to if the fisher cuts their catch but not in the are identified by the factor name, next is if the fisher cuts the catch in the anus (ACut), dorsal (DCut), mouth (MCut) and/or ventral (VCut) regions

**Boil.F, Boil.S & Boil.T:** Covariates coding the minutes spent boiling the catch in the first (Boil.F), second (Boil.S) and third (Boil.T) boils
