# Supplementary material for: Multiple Factors Affect Socioeconomics and Wellbeing of Artisanal Sea Cucumber Fishers
Source: PLoS One. 2016 Dec 8;11(12):e0165633. doi: 10.1371/journal.pone.0165633 (PMC5145150; doi:10.1371/journal.pone.0165633)
Supplement: S2 Table — (DOCX) [file pone.0165633.s002.docx]

**Supporting Information S2**

**Table S2**. **Prices per kilogram in AUD for dried sea cucumbers from different countries.** Price are current at the dates of the studies.

| **Species** | **Kenya 2004 [1]** ^†^ | **Solomon Islands 2005 [2]** ^‡^ | **Yemen 2007 [3]**^⊗^ | **Madagascar 2007 [4]**^φ^ | **Vietnam 2005 [5]**^∞^ | **Kiribati 2011 (this study) (mean, max)**** | **Fiji 2014 (this study) (mean, max)**** |
| --- | --- | --- | --- | --- | --- | --- | --- |
| Snakefish |  | 9 |  |  |  |  | 16, 35 |
| Pinkfish |  | 6 |  |  | 4 | 9, 35 | 7, 17 |
| Lollyfish |  | 6 | 18 |  | 3 | 4, 7 | 7, 17 |
| Black teatfish |  | 29 |  |  |  | 39, 65 | 42, 104 |
| White teatfish | 39 | 43 | 37 | 59 |  | 46, 65 | 73, 122 |
| Elephant trunkfish |  | 6 |  |  | 7 | 18, 35 | 9, 20 |
| Stonefish | 6 | 37 |  | 12 |  | 36, 35 | 36, 87 |
| Hairy blackfish |  | 37 |  |  |  | 23, 17 | 32, 87 |
| Surf redfish |  | 37 | 18 | 12 |  | 22, 36 | 28, 58 |
| Leopardfish |  | 14 |  |  | 25 | 15, 35 | 19, 41 |
| Chalkfish |  |  |  |  | 8 | 17, 17 | 12, 35 |
| Brown Sandfish |  | 14 |  |  |  | 15, 35 | 12, 35 |
| Greenfish |  | 42 |  |  | 25 | 29, 45 | 45, 87 |
| Curryfish | 11 | 37 |  | 20 | 25 | 48, 35 | 28, 70 |
| Prickly redfish | 28 | 38 |  | 23 | 66 | 38, 60 | 47, 93 |
| Sandfish |  |  | 24 | 59 | 58 |  | 20, 46 |
| Golden sandfish |  |  |  |  |  |  | 25, 29 |
| Deepwater redfish | 8 | 38 |  | 12 |  | 27, 20 | 37, 87 |
| Panning’s blackfish |  |  |  |  |  | 13, 35 | 56, 87 |
| Dragonfish |  | 37 |  |  |  | 7 | 27, 58 |
| Flowerfish |  |  |  |  |  | 30, 45 | 8, 23 |
| Amberfish | 2 | 9 | 29 |  |  | 23, 20 | 18, 35 |

^†^ Maximum of reported price range. 2004 exchange rate: 1 KSH = 0.014 AUD.

^‡^ Averages of prices from Kia and Honiara for large or A-grade product. 2005 exchange rate: 1 SBD = 0.18 AUD.

^⊗^ 2007 exchange rate: 1 USD = 1.22 AUD

^φ^ 2007 exchange rate: 1 MGA = 0.00065 AUD

^∞^ 2005 exchange rate: 1 VND = 0.000083 AUD

** Based on prices for large individuals

**References**

1. Ochiewo J, de la Torre-Castro M, Muthama C, Munyi F, Nthuta JM. Socio-economic features of sea cucumber fisheries in southern coast of Kenya. Ocean Coast Manage. 2010; 53(4): 192-202.

2. Ramofafia C, Nash W, Sibiti S, Makini D, Schwarz AM. Household socio-economics and bêche-de-mer resource use in Kia community, Isabel Province, Solomon Islands (June 2005). Gizo, Solomon Islands: WorldFish Center, 2007.

3. Lindsay S, Al-Agwan Z. Sea cucumber fisheries of Yemen: status and recommendations. Jeddah, Saudi Arabia: PERSGA, 2009.

4. Lavitra T, Rachelle D, Rasolofonirina R, Jangoux M, Eeckhaut I. Processing and marketing of holothurians in the Toliara region, southwestern Madagascar. SPC Beche-de-mer Inf Bull. 2008; 28: 24-33.

5. del Mar Otero-Villanueva M, Ut VN. Sea cucumber fisheries around Phu Quoc Archipelago: A cross-border issue between South Vietnam and Cambodia. SPC Beche-de-mer Inf Bull. 2007; 25: 32-6.
