# Supplementary material for: Multiple Factors Affect Socioeconomics and Wellbeing of Artisanal Sea Cucumber Fishers
Source: PLoS One. 2016 Dec 8;11(12):e0165633. doi: 10.1371/journal.pone.0165633 (PMC5145150; doi:10.1371/journal.pone.0165633)
Supplement: S3 Table — (DOCX) [file pone.0165633.s003.docx]

**Supporting Information S3**

**Table S3.** **Prices per piece (individual animal) in AUD for fresh sea cucumbers from different countries.** Prices are current at the dates of the studies.

| **Species** | **Madagascar 2007 [1]** ^†^ | **Sri Lanka 2008–2009 [2]** ^‡^ | **Zanzibar 2009 [3]** ^φ^ | **Philippines 2010 [4]*** | **Kiribati 2011 (this study) (mean, max)**** | **Tonga 2011 (this study) (mean, max)**** | **Fiji 2014 (this study) (mean, max)**** |
| --- | --- | --- | --- | --- | --- | --- | --- |
| Snakefish |  |  | 0.1 |  |  |  | 1.0, 3 |
| Pinkfish |  |  |  |  | 0.9, 0.8 | 2 | 1, 8 |
| Lollyfish |  | 0.7 | 0.1 | 0.02 | 0.5, 0.5 | 0.3 | 0.7, 7 |
| Black teatfish |  |  |  |  | 8, 12 | 23, 39 | 11, 40 |
| White teatfish | 3.3 | 15 |  |  | 12, 20 | 27, 43 | 34, 87 |
| Elephant trunkfish |  |  | 0.5 |  | 2, 2 | 0.8, 2 | 3, 9 |
| Stonefish | 0.2 |  | 0.2 |  | 1.2 | 4, 11 | 7, 15 |
| Hairy blackfish |  | 6 | 2 |  |  | 5, 9 | 6, 15 |
| Surf redfish | 0.2 |  | 0.2 |  | 1.4, 3 | 3, 5 | 4, 20 |
| Leopardfish |  |  |  |  | 1.1, 2 | 1, 2 | 3, 9 |
| Chalkfish |  | 0.6 |  |  | 0.7, 0.6 | 1, 2 | 2, 4 |
| Brown Sandfish |  | 3 | 1 |  | 1.0, 1.5 | 0.9, 2 | 2, 7 |
| Greenfish |  | 0.7 |  |  | 1.0, 2 |  | 0.6, 2 |
| Curryfish | 0.8 |  | 2 |  | 6, 5 | 2, 5 | 4, 12 |
| Prickly redfish | 3.3 |  | 8 |  | 8, 10 | 6, 11 | 9, 23 |
| Sandfish | 2.6 | 12 | 4 |  |  |  | 3, 7 |
| Golden sandfish |  |  | 7 |  |  | 7, 7 | 3, 7 |
| Deepwater redfish | 0.2 | 6 | 0.2 | 0.1 |  | 3, 4 | 7, 15 |
| Panning’s blackfish |  |  |  |  | 4, 5 | 6, 8 | 6, 12 |
| Dragonfish | 0.8 |  |  |  |  | 2, 2 | 2, 5 |
| Flowerfish |  |  |  |  |  |  | 2, 7 |
| Amberfish |  | 3 | 2 |  | 0.5 | 1, 4 | 3, 6 |

^†^ Upper price of reported value range. 2007 exchange rate: 1 MGA = 0.00065 AUD.

^‡^2009 exchange rate: 1 USD = 1.31 AUD

^φ^ Upper price of reported value range. 2009 exchange rate 1 TZS = 0.00098 AUD.

* 2009 exchange rate: 1 PHP = 0.024 AUD

** Based on prices for large individuals.

**References**

1. Lavitra T, Rachelle D, Rasolofonirina R, Jangoux M, Eeckhaut I. Processing and marketing of holothurians in the Toliara region, southwestern Madagascar. SPC Beche-de-mer Inf Bull. 2008; 28: 24-33.

2. Dissanayake DCT, Stefansson G. Present status of the commercial sea cucumber fishery off the north-west and east coasts of Sri Lanka. J Mar Biol Assoc U K. 2012; 94(4): 831–41.

3. Eriksson HB, de la Torre-Castro M, Eklöf J, Jiddawi N. Resource degradation of the sea cucumber fishery in Zanzibar, Tanzania: a need for management reform. Aquat Living Resour. 2010; 23(04): 387-98.

4. Brown EO, Perez ML, Garces LR, Ragaza RJ, Bassig RA, Zaragoza EC. Value chain analysis for sea cucumber in the Philippines. Penang: The WorldFish Center; 2010. 44 p.
